# The Cause of Addison’s Disease

**Published:** 1897-01

**Authors:** 


THE CAUSE OF ADDISON'S DISEASE. Since this malady was first clearly defined as a pathological entity its cause has continued a mystery ; but the problem of its causation seems to have been recently solved by a discovery made at the Pathological Institute of Berlin, viz., that a brown-coloring matter known as pyrocatechin or brenzcatechin is secreted or formed by the suprarenal capsules. Its production is confined to the medullary substance of the glands. This peculiar substance quickly turns brown by contact with the air, which explains the brown pigment present in Addison's disease. Investigation of its properties shows the pyrocatechin to be a poison, which leads to the natural conclusion that it may be responsible not only for the pigmentation present in Addison's disease, but for the other symptoms characteristic of this malady.—*Mod. Med. and Bacteriological Review.*

PROFESSOR VON KOLLIKEE recommends a new preparation derived from iodoform and paraldehyde, and known as iodoformin, as a substitute for iodoform. It is odorless ; it can be sterilized by heat, and does not produce eczema from irritation of the tissues.—*Ibid.*

E. BAUMANN has recently shown that the thyroid gland contains iodine. His experiments led him to suspect that in diseased conditions this gland contains less iodine than in a normal state.—*Ibid.*

SELIGSON states that if the right ovary is removed from a rabbit the animal bears afterward only female young, while if the left ovary is removed the animal bears only male young. In nineteen cases of tubal pregnancy the fœtus when found upon the right side was a male, when upon the left side always a female.—*Ibid.*

MARSHALL recommends dipping instruments, after cleansing, in 20 per cent. solution of bicarbonate of soda and alcohol. It is stated that steel, iron, and copper instruments may be kept bright in this way.—*Ibid.*

MM. GILBERT and ROGER'S experiments recently published show conclusively that the tuberculosis of human beings and of birds is one and the same disease. Observations made by Cadiot and Strauss also confirm this fact.—*Ibid.*

DR. CHARRIN has recently reported to the Biological Society of Paris some observations made by Dr. Pettit which go to confirm, what has previously been believed, that at least one of the functions of suprarenal capsules is the destruction of poisons, especially those produced by microbes.—*Ibid.*

A CRITICISM of the medical press and its tendency to run toward the lower animals in the efforts to find originating causes of many infectious and contagious diseases is the result of a suggestion of German origin that measles of man may be conveyed to the pig, and in the latter be transmitted to others, and perhaps that some alleged outbreaks of swine-disease may only be measles.—*Veterinary Record*, December.

RABIES in Ireland continues to be a source of disease of much concern, as the statistics show large numbers affected.—*Ibid.*

SOUTH AFRICA has sought the services of Professor Koch as to the best methods of dealing with rinderpest.—*Ibid.*

IN a series of cases cannabis indica or Indian hemp in doses ranging from one drachm to an ounce and a half, while somewhat uncertain in action at times, was eminently satisfactory in a large variety of colicky cases. The safest dose ranged from one to four drachms, repeated if necessary. Small doses rarely produced the objectionable constipation that followed large doses. After the exhilarating stage dulness and stupor followed in all cases, lasting a variable period of time, according to the amount given. Lessened pulse and respiration, and sometimes a subnormal temperature, drawn condition of the penis, and lessened activity of the bowels were the dominant actions produced.—*Ibid.*

